# Osteomyelitis Due to *Mycobacterium goodii* in an Adolescent, United States

**DOI:** 10.3201/eid2611.200206

**Published:** 2020-11

**Authors:** Alejandro Diaz, Monica I. Ardura, Huanyu Wang, Stella Antonara, Christopher P. Ouellette

**Affiliations:** Nationwide Children’s Hospital and The Ohio State University, Columbus, Ohio, USA (A. Diaz, M.I. Ardura, H. Wang, C.P. Ouellette);; Riverside Methodist Hospital, Columbus (S. Antonara)

**Keywords:** Nontuberculous mycobacteria, Mycobacterium goodii, pediatric, osteomyelitis, bacteria, tuberculosis and other mycobacteria, antimicrobial resistance, United States

## Abstract

Osteomyelitis is a rare clinical manifestation of infection with nontuberculous mycobacteria (NTM). We report an adolescent with femoral osteomyelitis associated with prosthetic material due to an emerging pathogen, *Mycobacterium goodii.* Application of *secA1* and 16S ribosomal RNA gene sequencing reliably determined the NTM species, enabling targeted antimicrobial therapy.

Nontuberculous mycobacteria (NTM) are an emerging cause of human infections, likely because of improved detection methods and an increasing high-risk population (*1*–*3*). Coventional methods to identify NTM species rely on phenotypic characteristics to differentiate the most common species, but these labor-intensive and time-consuming methods delay final identification and appropriate therapy (*2*). Sequencing of 16S rRNA and *secA1* (essential secretory protein SecA1) genes provides an accurate and cost-effective method for NTM identification, offering a turnaround time of 1–2 days compared with 2–6 weeks for results from conventional methods (*4*).

*M. goodii* is a rapidly growing mycobacterium that can be nonpigmented or late-pigmented. Before 1999, the original classification of the 3 species in the *M. smegmatis* group identified 28 isolates of *M. goodii*, which most often were associated with posttraumatic wound infections (*5*). Since then, *M. goodii* has been implicated in infections related to prosthetic devices and penetrating trauma. Three recent reports detail 19 cases of *M. goodii* infections in patients with a mean age of 60 years (range 6–85 years). Types of infection included prosthetic device or pocket infection (n = 12), wound infection (n = 3), endocarditis (n = 1), pneumonia (n = 2), and endophthalmitis (n = 1) (*6*–*8*). We noted only 3 pediatric cases in the literature: 2 cases of pneumonia, 1 in a 15-year-old girl and 1 in a 7-week-old infant; and 1 soft tissue infection in a 6-year-old boy (*6*,*9*,*10*). 

We report a 15-year-old male patient with severe bilateral knee flexion contractures who underwent bilateral femoral extension osteotomies with hardware implantation. Two months later, he had intermittent low-grade fevers, right thigh pain, and surgical wound dehiscence with drainage. Initial laboratory results showed elevated leukocyte count, erythrocyte sedimentation rate, and C-reactive protein (Figure). An incision and discharge was performed but the femoral hardware was retained; 4 days later, a second incision and drainage was performed with primary closure. 

**Figure:**
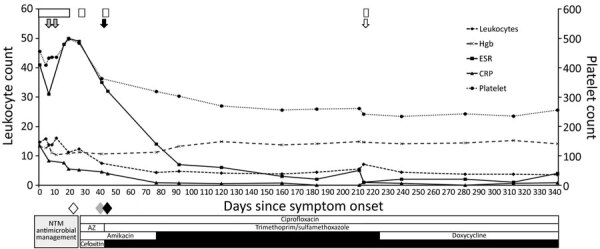
Timeline of laboratory values, surgical interventions, notification of pertinent culture results, and antimicrobial drug therapy in a case of osteomyelitis due to *Mycobacterium goodii *in an adolescent, United States. White boxes represent periods of hospitalization. Gray arrows indicate dates of surgical intervention with cultures obtained and femoral hardware retained. Solid black arrow indicates surgical intervention with cultures obtained and femoral hardware removed. Shaded arrow indicates interventional radiology aspiration of subcutaneous fluid collection with cultures obtained. Shaded diamond indicates first notification of nontuberculous mycobacterium growth. Solid gray diamond indicates first notification of *M. goodii* by 16S and *secA1* sequencing. Solid black diamond indicates first notification of susceptibility results for *M. goodii. *AZ, azithromycin; CRP, C-reactive protein; ESR, erythrocyte sedimentation rate; Hgb, hemoglobin; NTM, nontuberculous mycobacteria.

Acid-fast bacillus (AFB) cultures were obtained; after 22 days, NTM growth was identified. Empiric therapy was initiated with intravenous (IV) amikacin (15 mg/kg 1×/d), IV cefoxitin (3,000 mg every 8 h), oral azithromycin (250 mg 1×/d), and oral ciprofloxacin (500 mg every 12 h). Two weeks later, the patient underwent a third incision and drainage and hardware was removed because of recrudescent fever and surgical site discharge. AFB tissue cultures from bone again grew NTM. Sequencing of the *secA1* and 16S genes from all NTM isolates identified *M. goodii* (Appendix Figure). The patient’s therapy was modified to oral trimethoprim/sulfamethoxazole (TMP/SMX; 320 mg 2×/d, 6 mg/kg/dose based on TMP component), with continued IV amikacin and oral ciprofloxacin. Antimicrobial susceptibility testing results confirmed susceptibility to TMP/SMX, ciprofloxacin, amikacin, and doxycycline but noted resistance to clarithromycin and cefoxitin. Amikacin was discontinued after a total of 36 days of therapy. 

Five months after his last surgical intervention, the patient had clear discharge from his right thigh. A small superficial skin abscess was noted on magnetic resonance imaging. Fine needle aspiration of the fluid collection was performed from which AFB cultures were sterile but universal bacterial 16S rDNA PCR detected *M. goodii.* Given the potential for antimicrobial resistance, oral doxycycline (100 mg 2×/d) was added to the patient’s antimicrobial drug regimen. The 3-drug regimen was continued for an additional 4 months. Repeat imaging at the end of therapy showed no evidence of fluid reaccumulation, and the patient has not had an infection relapse for 10 months after discontinuation of antimicrobial drug therapy. No other *M. goodii* infections have been identified at our institution since this case.

NTM osteomyelitis treatment can be challenging. Management strategies include prolonged antimicrobial drug therapy, surgical debridement, and removal of foreign material (*2*). *M. goodii* usually is susceptible to TMP/SMX, amikacin, ciprofloxacin, imipenem, and doxycycline (*5*). However, *M. goodii* is intrinsically resistant to macrolides and rifampin, which commonly are used for empirical therapy of NTM infections; early species identification is crucial to ensuring effective and timely treatment (*2*,*5*,*6*). Optimal treatment is unknown, but a combination of >2 active drugs, for a minimum of 6 months, combined with surgical debridement and hardware removal, is recommended to ensure clinical and bacteriological cure and prevent antimicrobial resistance (*2*,*6*). 

Our case shows similarities to prior adult reports, specifically prosthetic-associated *M. goodii* infection, and further highlights the emergence of this pathogen in the pediatric population. Given the repeated culture-positive results from our patient, we do not believe this case was the result of an environmental contaminant. In addition, no other cases of *M. goodii* infection have been identified at our institution to suggest nosocomial infection, but we cannot definitively exclude this mode of acquisition. 

In conclusion, our case highlights *M. goodii* as an emerging pediatric NTM pathogen. These findings underscore the use of *secA1* and 16S rRNA sequencing for rapid species identification to enable timely and effective antimicrobial drug therapy.

Appendix Phylogenetic tree of mycobacterial isolates by *secA1* and partial 16S sequences from a case of osteomyelitis in an adolescent caused by *Mycobacterium goodii *and sequences from publicly available databases.

## References

[1] Brown-Elliott BA, Wallace RJ Jr. Clinical and taxonomic status of pathogenic nonpigmented or late-pigmenting rapidly growing mycobacteria. Clin Microbiol Rev. 2002;15:716–46. 10.1128/CMR.15.4.716-746.2002 12364376 PMC126856

[2] Griffith DE, Aksamit T, Brown-Elliott BA, Catanzaro A, Daley C, Gordin F, et al.; ATS Mycobacterial Diseases Subcommittee; American Thoracic Society; Infectious Disease Society of America. An official ATS/IDSA statement: diagnosis, treatment, and prevention of nontuberculous mycobacterial diseases. Am J Respir Crit Care Med. 2007;175:367–416. 10.1164/rccm.200604-571ST 17277290

[3] Tebruegge M, Pantazidou A, MacGregor D, Gonis G, Leslie D, Sedda L, et al. Nontuberculous Mycobacterial Disease in Children - Epidemiology, Diagnosis & Management at a Tertiary Center. PLoS One. 2016;11:e0147513. 10.1371/journal.pone.0147513 26812154 PMC4727903

[4] Cook VJ, Turenne CY, Wolfe J, Pauls R, Kabani A. Conventional methods versus 16S ribosomal DNA sequencing for identification of nontuberculous mycobacteria: cost analysis. J Clin Microbiol. 2003;41:1010–5. 10.1128/JCM.41.3.1010-1015.2003 12624023 PMC150297

[5] Brown BA, Springer B, Steingrube VA, Wilson RW, Pfyffer GE, Garcia MJ, et al. *Mycobacterium wolinskyi* sp. nov. and *Mycobacterium goodii* sp. nov., two new rapidly growing species related to *Mycobacterium smegmatis* and associated with human wound infections: a cooperative study from the International Working Group on Mycobacterial Taxonomy. Int J Syst Bacteriol. 1999;49:1493–511. 10.1099/00207713-49-4-1493 10555330

[6] Salas NM, Klein N. *Mycobacterium goodii:* an emerging nosocomial pathogen: a case report and review of the literature. [Baltim Md]. Infect Dis Clin Pract (Baltim Md). 2017;25:62–5. 10.1097/IPC.0000000000000428 28286403 PMC5331875

[7] Harper G, Ong JL, Costanigro L. Oh Goody! Two additional *Mycobacterium goodii* infections. Open Forum Infectious Diseases. 2015;2(suppl_1):576.

[8] Parikh RB, Grant M. *Mycobacterium goodii* endocarditis following mitral valve ring annuloplasty. Ann Clin Microbiol Antimicrob. 2017;16:14. 10.1186/s12941-017-0190-4 28327156 PMC5361780

[9] Goussard P, Rabie H, Morrison J, Schubert PT. Superinfection with *Mycobacteria goodii* in a young infant with exogenous lipoid pneumonia. Pediatr Pulmonol. 2019;54:1345–7. 10.1002/ppul.24355 31206238

[10] Hougas JE III, Bruneteau RJ, Varman M. *Mycobacterium goodii* infection of skin graft in an immunocompetent child. Infect Dis Clin Pract. 2011;19:146–7. 10.1097/IPC.0b013e3182002df1

