# Supplementary material for: Osteomyelitis Due to Mycobacterium goodii in an Adolescent, United States
Source: Emerg Infect Dis. 2020 Nov;26(11):2781–3. doi: 10.3201/eid2611.200206 (PMC7588509; doi:10.3201/eid2611.200206)
Supplement: Appendix — Phylogenetic tree of mycobacterial isolates by secA1 and partial 16S sequences from a case of osteomyelitis in an adolescent caused by Mycobacterium goodii and sequences from publicly available databases. [file 20-0206-Techapp-s1.pdf]

Article DOI: https://doi.org/10.3201/eid2611.200206

Osteomyelitis Due to *Mycobacterium goodii* in an Adolescent, United States

Appendix

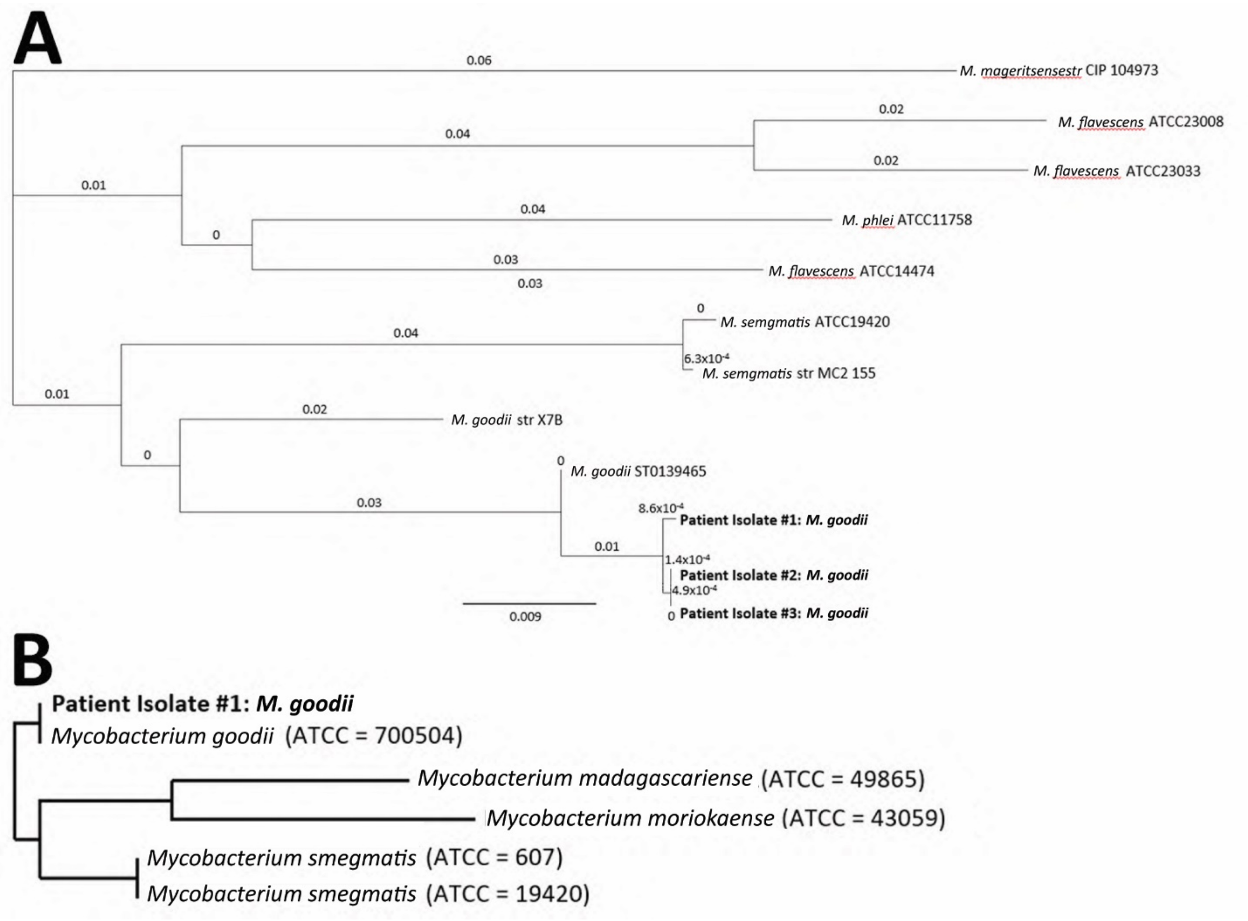

Appendix Figure. Phylogenetic tree of mycobacterial isolates by *secA1* and partial 16S sequences comparing for *Mycobacterium goodii* from a case of osteomyelitis to other species. A) Phylogenetic tree of partial *secA1* sequences of various mycobacterial species. The phylogenetic tree is derived from *secA1* sequences from 7 American Type Culture Collection (ATCC; https://www.atcc.org) mycobacterial strains that are closely related to *M. goodii*, 2 *M. goodii secA1* sequences deposited in the National Center for Biotechnology Information database (NCBI; https://www.ncbi.nlm.nih.gov), and the *M. goodii* isolates from

the patient in this report. The tree created by the *secA1* sequences reflects the discriminatory power of the assay to differentiate among various species that are grouped together by other assays (e.g., HPLC and 16S sequencing). B) Phylogenetic tree of partial 16S sequences of closely related mycobacteria. The phylogenetic tree is derived from partial 16S sequences from 5 ATCC mycobacterial strains that are closely related to *M. goodii* and the 16S sequence of the *M. goodii* isolate from the patient in this report. It reflects the discriminatory power of the sequencing assay to differentiate among various species of mycobacteria. Bold text indicates isolates from patient in this report. Scale bar indicates nucleotide substitutions per site.
